# Supplementary material for: HIVprotI: an integrated web based platform for prediction and design of HIV proteins inhibitors
Source: J Cheminform. 2018 Mar 9;10:12. doi: 10.1186/s13321-018-0266-y (PMC5845081; doi:10.1186/s13321-018-0266-y)
Supplement: Supplementary file 2 — Additional file 2. Source code of HIVProtI web server. [file 13321_2018_266_MOESM2_ESM.zip › HIVprotI_Source-code/hivprotI/weka-3-6-6/doc/weka/core/DistanceFunction.html]

DistanceFunction


---


|  |  |  |
| --- | --- | --- |
| **Overview** \| **Package** \| **Class** \| **Tree** \| **Deprecated** \| **Index** \| **Help** \| **Weka's home** | |  |
| **PREV CLASS**   **NEXT CLASS** | **FRAMES**    **NO FRAMES**     **All Classes** |  |
| SUMMARY: NESTED \| FIELD \| CONSTR \| METHOD | DETAIL: FIELD \| CONSTR \| METHOD |  |


---


## weka.core Interface DistanceFunction

**All Superinterfaces:**: OptionHandler

**All Known Implementing Classes:**: AbstractStringDistanceFunction, ChebyshevDistance, EditDistance, EuclideanDistance, ManhattanDistance, NormalizableDistance

---

``` public interface DistanceFunction extends OptionHandler ```

Interface for any class that can compute and return distances between two
instances.

**Version:**
:   $Revision: 1.7 $

**Author:**
:   Ashraf M. Kibriya (amk14@cs.waikato.ac.nz)

---

| **Method Summary** | |
| --- | --- |
| `double` | `distance(Instance first, Instance second)`             Calculates the distance between two instances. |
| `double` | `distance(Instance first, Instance second, double cutOffValue)`             Calculates the distance between two instances. |
| `double` | `distance(Instance first, Instance second, double cutOffValue, PerformanceStats stats)`             Calculates the distance between two instances. |
| `double` | `distance(Instance first, Instance second, PerformanceStats stats)`             Calculates the distance between two instances. |
| `java.lang.String` | `getAttributeIndices()`             Gets the range of attributes used in the calculation of the distance. |
| `Instances` | `getInstances()`             returns the instances currently set. |
| `boolean` | `getInvertSelection()`             Gets whether the matching sense of attribute indices is inverted or not. |
| `void` | `postProcessDistances(double[] distances)`             Does post processing of the distances (if necessary) returned by distance(distance(Instance first, Instance second, double cutOffValue). |
| `void` | `setAttributeIndices(java.lang.String value)`             Sets the range of attributes to use in the calculation of the distance. |
| `void` | `setInstances(Instances insts)`             Sets the instances. |
| `void` | `setInvertSelection(boolean value)`             Sets whether the matching sense of attribute indices is inverted or not. |
| `void` | `update(Instance ins)`             Update the distance function (if necessary) for the newly added instance. |

**Methods inherited from interface weka.core.OptionHandler**

`getOptions, listOptions, setOptions`

**Method Detail**

### setInstances

```
void setInstances(Instances insts)
```

:   Sets the instances.

    :   **Parameters:**: `insts` - the instances to use

---


### getInstances

```
Instances getInstances()
```

:   returns the instances currently set.

    :   **Returns:**: the current instances

---


### setAttributeIndices

```
void setAttributeIndices(java.lang.String value)
```

:   Sets the range of attributes to use in the calculation of the distance.
    The indices start from 1, 'first' and 'last' are valid as well.
    E.g.: first-3,5,6-last

    :   **Parameters:**: `value` - the new attribute index range

---


### getAttributeIndices

```
java.lang.String getAttributeIndices()
```

:   Gets the range of attributes used in the calculation of the distance.

    :   **Returns:**: the attribute index range

---


### setInvertSelection

```
void setInvertSelection(boolean value)
```

:   Sets whether the matching sense of attribute indices is inverted or not.

    :   **Parameters:**: `value` - if true the matching sense is inverted

---


### getInvertSelection

```
boolean getInvertSelection()
```

:   Gets whether the matching sense of attribute indices is inverted or not.

    :   **Returns:**: true if the matching sense is inverted

---


### distance

```
double distance(Instance first,
                Instance second)
```

:   Calculates the distance between two instances.

    :   **Parameters:**: `first` - the first instance: `second` - the second instance **Returns:**: the distance between the two given instances

---


### distance

```
double distance(Instance first,
                Instance second,
                PerformanceStats stats)
                throws java.lang.Exception
```

:   Calculates the distance between two instances.

    :   **Parameters:**: `first` - the first instance: `second` - the second instance: `stats` - the performance stats object **Returns:**: the distance between the two given instances **Throws:**: `java.lang.Exception` - if calculation fails

---


### distance

```
double distance(Instance first,
                Instance second,
                double cutOffValue)
```

:   Calculates the distance between two instances. Offers speed up (if the
    distance function class in use supports it) in nearest neighbour search by
    taking into account the cutOff or maximum distance. Depending on the
    distance function class, post processing of the distances by
    postProcessDistances(double []) may be required if this function is used.

    :   **Parameters:**: `first` - the first instance: `second` - the second instance: `cutOffValue` - If the distance being calculated becomes larger than cutOffValue then the rest of the calculation is discarded. **Returns:**: the distance between the two given instances or Double.POSITIVE\_INFINITY if the distance being calculated becomes larger than cutOffValue.

---


### distance

```
double distance(Instance first,
                Instance second,
                double cutOffValue,
                PerformanceStats stats)
```

:   Calculates the distance between two instances. Offers speed up (if the
    distance function class in use supports it) in nearest neighbour search by
    taking into account the cutOff or maximum distance. Depending on the
    distance function class, post processing of the distances by
    postProcessDistances(double []) may be required if this function is used.

    :   **Parameters:**: `first` - the first instance: `second` - the second instance: `cutOffValue` - If the distance being calculated becomes larger than cutOffValue then the rest of the calculation is discarded.: `stats` - the performance stats object **Returns:**: the distance between the two given instances or Double.POSITIVE\_INFINITY if the distance being calculated becomes larger than cutOffValue.

---


### postProcessDistances

```
void postProcessDistances(double[] distances)
```

:   Does post processing of the distances (if necessary) returned by
    distance(distance(Instance first, Instance second, double cutOffValue). It
    may be necessary, depending on the distance function, to do post processing
    to set the distances on the correct scale. Some distance function classes
    may not return correct distances using the cutOffValue distance function to
    minimize the inaccuracies resulting from floating point comparison and
    manipulation.

    :   **Parameters:**: `distances` - the distances to post-process

---


### update

```
void update(Instance ins)
```

:   Update the distance function (if necessary) for the newly added instance.

    :   **Parameters:**: `ins` - the instance to add


---


|  |  |  |
| --- | --- | --- |
| **Overview** \| **Package** \| **Class** \| **Tree** \| **Deprecated** \| **Index** \| **Help** \| **Weka's home** | |  |
| **PREV CLASS**   **NEXT CLASS** | **FRAMES**    **NO FRAMES**     **All Classes** |  |
| SUMMARY: NESTED \| FIELD \| CONSTR \| METHOD | DETAIL: FIELD \| CONSTR \| METHOD |  |


---
